# From extraocular photoreception to pigment movement regulation: a new control mechanism of the lanternshark luminescence

**DOI:** 10.1038/s41598-020-67287-w

**Published:** 2020-06-23

**Authors:** Laurent Duchatelet, Tomohiro Sugihara, Jérôme Delroisse, Mitsumasa Koyanagi, René Rezsohazy, Akihisa Terakita, Jérôme Mallefet

**Affiliations:** 1 Université Catholique de Louvain (UCLouvain), Marine Biology Laboratory, Earth and Life Institute, 3 Croix du Sud, Louvain-La-Neuve, 1348 Belgium; 2 Osaka City University, Department of Biology and Geosciences, Graduate School of Science, Osaka, 558-8585 Japan; 3 Université de Mons (UMONS), Biology of Marine Organisms and Biomimetics, Research Institute for Biosciences, 23 Place du Parc, 7000 Mons, Belgium; 4 Université Catholique de Louvain (UCLouvain), Animal Molecular and Cellular Biology, Louvain Institute of Biomolecular Science and Technology, 5 Croix du Sud, Louvain-la-Neuve, 1348 Belgium

**Keywords:** Physiology, Hormone receptors, Phosphoinositol signalling, Motor proteins, Animal physiology, Ichthyology

## Abstract

The velvet belly lanternshark, *Etmopterus spinax*, uses counterillumination to disappear in the surrounding blue light of its marine environment. This shark displays hormonally controlled bioluminescence in which melatonin (MT) and prolactin (PRL) trigger light emission, while α-melanocyte-stimulating hormone (α-MSH) and adrenocorticotropic hormone (ACTH) play an inhibitory role. The extraocular encephalopsin (Es-Opn3) was also hypothesized to act as a luminescence regulator. The majority of these compounds (MT, α-MSH, ACTH, opsin) are members of the rapid physiological colour change that regulates the pigment motion within chromatophores in metazoans. Interestingly, the lanternshark photophore comprises a specific iris-like structure (ILS), partially composed of melanophore-like cells, serving as a photophore shutter. Here, we investigated the role of (*i*) Es-Opn3 and (*ii*) actors involved in both MT and α-MSH/ACTH pathways on the shark bioluminescence and ILS cell pigment motions. Our results reveal the implication of Es-Opn3, MT, inositol triphosphate (IP_3_), intracellular calcium, calcium-dependent calmodulin and dynein in the ILS cell pigment aggregation. Conversely, our results highlighted the implication of the α-MSH/ACTH pathway, involving kinesin, in the dispersion of the ILS cell pigment. The lanternshark luminescence then appears to be controlled by the balanced bidirectional motion of ILS cell pigments within the photophore. This suggests a functional link between photoreception and photoemission in the photogenic tissue of lanternsharks and gives precious insights into the bioluminescence control of these organisms.

## Introduction

Camouflage is one of the most powerful anti-predatory tools on earth^1^. By mimicking the colour of the environment background, many organisms successfully escape predation^1,2^. An efficient camouflage strategy needs two essential and interconnected mechanisms: (*i*) an accurate sensory machinery to evaluate the environment and (*ii*) the genetic determination for expressing a phenotypic trait mimicking the environment or/and the capability to modulate the skin colouration to match with the background colour. Countershading, a type of camouflage strategy which consists of the gradation of colour from dark on the dorsal side to light on the ventral area, is generally considered as an efficient hiding strategy spread mainly in the marine environment^1–4^. The cryptic strategy aims to facilitate the concealment of the projected shadow by the body adding a clear betterment to the organism’s survival^5^. This mechanism may be passive, with no colour modification during the organism life, or active, with the ability to gradually modify the skin colour to adapt the background colour (*i.e*. in terms of dark-grey scale or colour).

Skin colour modifications need to be under fine-tuned modulation to display an efficient camouflage. Pathways controlling colour modifications involve the motion of pigmented granule (*i.e*. aggregation and dispersion). These processes appeared to be conserved across metazoans with common use of multiple molecular actors (*e.g*. melatonin, melanocortin, prolactin, γ-aminobutyric acid, calcineurin, cyclic adenosine monophosphate, inositol triphosphate, dynein, kinesin, extraocular opsins, melanin) in a large diversity of organisms^6–16^. Both pigment dispersion and aggregation in metazoan melanophores are described as microtubule-dependent processes^17–20^. In the epipelagic marine environment (*i.e*. between 0 and 200 m depth), many cephalopods^21–25^ and fishes^26–28^ use camouflage to avoid being spotted by predators. Chromatic countershading camouflage type may also be used in the same oceanic layer as predation support as exemplified in the tiger shark^29^. Even in the mesopelagic zone (*i.e*. between 200 and 1000 m depth), where a faint blue light remains^30–32^, a similar function was evolutionary put forward allowing bioluminescent organisms to hide from predation^33–36^. This countershading mechanism, the counterillumination, is achieved when a luminous organism ventrally emits a light that mimics the one in the surrounding environment in terms of wavelength, intensity and angular distribution to avoid being seen by underneath swimming predators^34,37–40^. Within mesopelagic bioluminescent organism communities, luminous sharks display this type of specific anti-predatory strategy.

**Figure 1:**
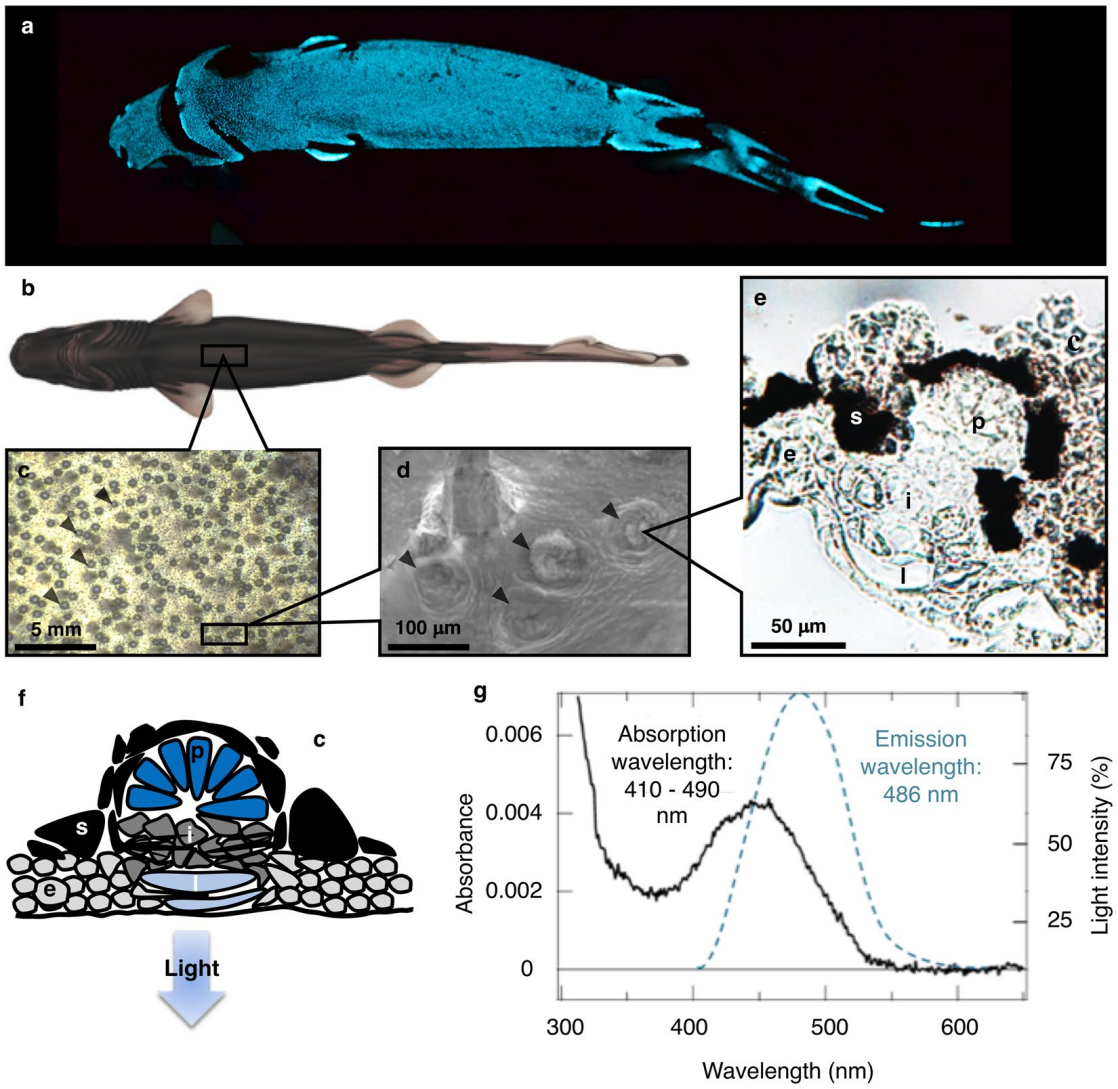
View of the light emission structure for *E. spinax*. (**a**) *E. spinax* ventral luminescence. (**b**) *E. spinax* schematic ventral view. (**c**) Ventral skin patch under optic microscope. (**d**) Close-up on photophores under scanning electron microscope. (**e**) Photophore cryosection. (**f**) Schematic representation of the photophore structure. (**g**) Graphical view of the *E. spinax* light emission spectrum and the absorption wavelength of the Es-Opn3. Arrowheads correspond to photophores. c: connective tissue; e: epidermis; i: iris-like structure cells; l: lens cells; p: photocytes; s: pigmented sheath.

Represented by at least 43 benthopelagic species^41,42^, lanternsharks (*i.e*. Etmopteridae) present a large number of known species displaying the ability to intrinsically emit light mainly ventrally to camouflage, such as *Etmopterus spinax*, *Etmopterus molleri*, *Etmopterus splendidus*, *Trigonognathus kabeyai*^43–47^. They display thousands of tiny organs, photophores, that emit blue-green light (Fig. 1a–d)^47–49^. Among Etmopteridae, the velvet belly lanternshark, *Etmopterus spinax* is the most studied lanternshark. This small benthopelagic shark species, up to 60 cm in total length, is widespread on the East Atlantic continental shelf from the North of Norway to the coast of middle west Africa as well as in the Mediterranean Sea at depth ranging from 85 to 785 m^50–53^. Although *E. spinax* is a relatively common species, data on its biology and life style is limited. Stomach content studies reveal a diet mainly composed of small decapods, euphausids, cephalopods and teleosts^54,55^. Due to their small sizes, Etmopteridae species are preyed by larger sharks such as *Echinorhinus cookei*, *Heptanchrias perlo* and *Dalatias licha*^49,56–59^. *E. spinax*, as other Etmopteridae species, forms sex-dependent aggregations and seems to undertake vertical migrations either to reproduce, or follow isolumes^47,51,60–62^. This ovoviviparous species has a potential triannual reproductive cycle with a one year gestation period, typical of Squalidae sharks^63^, and an estimated lifespan of around 20 years^64^, with an appearance of sexual maturity between the fourth and the fifth year^63^. Most litters range from 10 to 12 pups (*i.e*. six pups in each uteri)^65^. The remaining literature on *E. spinax* ecological traits mainly deal with parasitism^66–68^ or highlight fishery issues^69,70^.

Histologically, *E. spinax* lanternshark photophores are composed of light-emitting cells, photocytes, encapsulated in a dark pigmented melanophore-like sheath, surmounted by a multilayer cell area, the iris-like structure (ILS), and topped by one or several lens cells (Fig. 1e,f)^71,72^. A fine regulation of light emission in bioluminescent organisms, such as this lanternshark, using light as an anti-predatory function is crucial to maximize their fitness. Recently, Claes and Mallefet (2009) and Duchatelet *et al*. (2020), described the actors involved in the intrinsic light emission control in *E. spinax*^44,48,73^. While melatonin (MT) and prolactin (PRL) trigger light emission, alpha-melanocyte-stimulating hormone (α-MSH) and adrenocorticotropic hormone (ACTH) inhibit luminescence^44,48^. Neuromodulators such as nitric oxide (NO) or γ-aminobutyric acid (GABA) also play a role in light emission control^74,75^. Interestingly, all these actors are essential and main players in the physiological skin colour change^9,10,13,14^.

Duchatelet *et al*. (2020), assumed a potential shift from pigment movement regulation pathway involved in whole skin camouflage in epipelagic sharks to bioluminescence regulation involved in counterillumination in luminous sharks^48^. They highlighted the implication of cAMP in the light emission control of Etmopteridae. Duchatelet *et al*. (2020), depicted the expression pattern and the co-localization of MT and α-MSH/ACTH receptors (MTNR and MCR, respectively), as well as the respective G protein within the light organ^76^. Furthermore, Delroisse *et al*. (2018) and Duchatelet *et al*. (2019) localized an encephalopsin (Es-opsin 3) within the photophores, and assumed the involvement of this extraocular opsin in the control of the lanternshark bioluminescence^77,78^. The encephalopsin is assumed to act as a light output feedback regulator within photophores. Previous studies already hypothesized that the extraocular opsins have implication in bioluminescence regulation in other taxa such as squid^79,80^, comb jelly^81^ or brittle star^82^.

All these researches support the implication of a mechanical control of bioluminescence through pigment movements within the ILS cells which act therefore as a light organ shutter. Nevertheless, to our knowledge, no study has examined the complete pathways involved in the phototransduction event (*i.e*. through lanternshark opsin 3), neither in the melatonin/melanocortin receptor transduction cascade leading the light emission control. Here, by step by step deciphering these two pathways, ILS shutter organ function is deciphered and clues are brought forward on an extraocular opsin implication in the light emission control of lanternshark luminous camouflage.

## Results

### *In vitro* characterization of the Opn3 photopigment

*In vitro* characterization of the Es-Opn3 was performed to examine its ability to perceive light and determine its absorption spectrum. The protein was expressed in COS1 mammalian cells and purified pigments were successfully obtained and characterized as a blue-sensitive pigment with an absorption spectrum ranging from 410 to 490 nm (Supplementary Fig. S1). Following Terakita *et al*. (2008), C-terminal truncated construction was also expressed in the mammalian cells^83^. The C-terminal truncation resulted in a more than 2-fold higher yield of the purified Es-Opn3-based pigment, which allowed to determine the maximum absorption of the Es-Opn3-based pigment at 445 nm (Fig. 1g).

### Light induced IP_3_ modulation within *E. spinax* photophores

**Figure 2:**
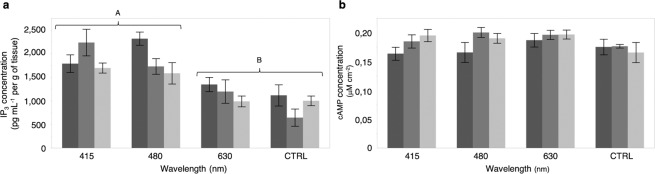
IP_3_ and cAMP concentration measurements according to exposure wavelength and time. (**a**) IP_3_ assays. (**b**) cAMP assays. Dark grey, medium grey, and light grey correspond to ventral skin patches enlightened during 15, 30 and 45 min, respectively. CTRL corresponds to skin patches kept in dark conditions. A and B represent statistically different groups.

IP_3_ is acting on IP_3_ receptors to mobilize intracellular Ca^2+^ that play a significant role in some photoresponse components. To determine the effect of light stimulations on photogenic skin (*i.e*. photophore region), putative modulation of IP_3_ concentrations after light exposure was evaluated on *E. spinax* ventral epidermis. Ventral skin patches enlighten with monochromatic light of 415 (bluish-violet), 480 (azure-blue) or 630 (orange-red) nm wavelengths presented a significant variation of the IP_3_ intracellular level (P < 0.05). *E. spinax* skin patches exposed during 15 min revealed an IP_3_ concentration level of 1787.7 ± 186.9 pg mL^−1^ at 415 nm, 2316.4 ± 139.4 pg mL^−1^ at 480 nm and 1345.6 ± 149.7 pg mL^−1^ at 630 nm (Fig. 2a). Thirty minutes exposure revealed a concentration of 2234.6 ± 280.5 pg mL^−1^ at 415 nm, 1728.4 ± 164.5 pg mL^−1^ at 480 nm and 1195.8 ± 248.8 pg mL^−1^ in red light (Fig. 2a). Tissues exposed during 45 min under 415 nm light presented an IP_3_ concentration of 1695.5 ± 105.5 pg mL^−1^; at 480 nm, a concentration of 1582.8 ± 225 pg mL^−1^ and at 630 nm, a concentration of 988.2 ± 113.6 pg mL^−1^ (Fig. 2a). Ventral skin patches preserved in dark condition were used as controls. They presented an IP_3_ intracellular level not significantly different from 630 nm experiments (P > 0.05); 15 min exposure led to an IP_3_ concentration level of 1115.7 ± 223.9 pg mL^−1^; 30 min, an IP_3_ concentration of 643.3 ± 182.3 pg mL^−1^ and 45 min, an IP_3_ concentration of 1000.7 ± 101.9 pg mL^−1^ (Fig. 2a). All values were expressed per gram of tissue. Therefore, *E. spinax* ventral skin, full of photophores, react to blue light wavelengths (*e.g*. similar to shark luminescence) by modulating the intracellular concentration of IP_3_.

### Absence of light-induced cAMP modulation within *E. spinax* photophores

Similarly to what was done for the IP_3_ concentration assays, to highlight the effect of light absorption (*e.g*. bioluminescence) at the level of photophores, cAMP concentration assays were performed after light exposure. *E. spinax* ventral skin patches enlightened during 15 min with wavelengths of 415, 480 or 630 nm did not show significant variation of the cAMP intracellular level (P > 0.05; Fig. 2b). The cAMP concentrations were 0.16 ± 0.01 µM cm^−2^ at 415 nm, 0.17 ± 0.02 µM cm^−2^ at 480 nm and 0.20 ± 0.01 µM cm^−2^ at 630 nm (Fig. 2b). Exposure of thirty minutes reveal concentrations of 0.18 ± 0.01 µM cm^−2^ at 415 nm, 0.20 ± 0.01 µM cm^−2^ at 480 nm and 0.20 ± 0.01 µM cm^−2^ at 630 nm (Fig. 2b). After 45 min of exposure, the cAMP concentrations were 0.20 ± 0.01 µM cm^−2^ at 415 nm; 0.19 ± 0.01 µM cm^−2^ at 480 nm and 0.20 ± 0.01 µM cm^−2^ at 630 nm (Fig. 2b). Controls (*i.e*. ventral skin patches preserved in dark condition) present a cAMP intracellular level that was not significantly different from the treatments (*i.e*. enlightened skin patches) (P > 0,05). After 15 min, a cAMP concentration of 0.18 ± 0.01 µM cm^−2^ was measured. After 30 and 45 min, cAMP concentrations of 0.18 ± 0.01 µM cm^−2^ and 0.17 ± 0.02 µM cm^−2^ were observed, respectively. Results are consistent with previous cAMP concentration measurements performed on Etmopteridae skin patches treated with various hormones^48^. Here, results demonstrated the non-involvement of cAMP in the phototransduction events.

### Iris-like structure regulation pathway

#### IP_3_ triggers photophore opening in a calcium dependent manner

**Figure 3:**
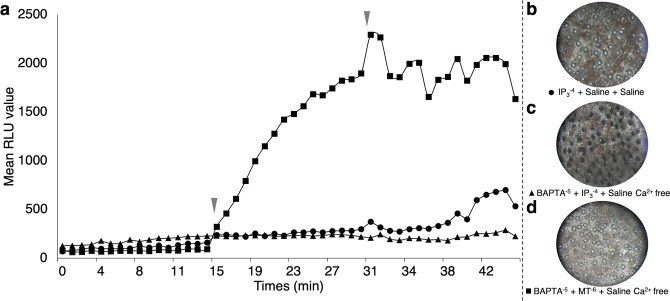
IP_3_ and calcium-related lumino-pharmacological assays. (**a**) mean time-course evolution of light emission following the three application treatments showing that melatonin does not need Ca^2+^ to trigger light emission, and an IP_3_ increase does not trigger light emission either in the presence or absence of calcium. Red arrowheads indicate the time of second and third drug applications. Final ventral skin patch pigmentation state after (**b**) D-myo-IP_3_ 10^−4^ mol l^−1^ + Shark saline + Shark saline applications showing an open state of photophores; (**c**) BAPTA 10^−5^ mol l^−1^ + D-myo-IP_3_ 10^−4^ mol l^−1^ + Saline Ca^2+^ free medium applications showing a close state of photophores; and (**d**) BAPTA 10^−5^ mol l^−1^ + MT 10^−6^ mol l^−1^ + Saline Ca^2+^ free medium applications showing an open state of photophores.

To determine the effect of an IP_3_ increase on the *E. spinax* light emission and photophore pigmentation state, application of D-*myo*-IP_3_ on ventral skin patches was performed. In parallel, effect of calcium absence was followed. IP_3_ was applied on freshly dissected ventral skin patches at a concentration of 10^−4^ M^84^. Alone, IP_3_ application does not trigger any light emission (Fig. 3a,b). The absence of light emission was also observed for IP_3_ application on skin patches in absence of free calcium (Fig. 3a,c). The pictures of the final pigmentation state of treated tissue samples revealed fully open photophores in the case of the IP_3_ treatment in presence of calcium (Fig. 3b). Conversely, the IP_3_ treatment in absence of calcium shown dark closed photophores (Fig. 3c). As a control, light emission was efficiently recorded after melatonin application in a calcium-free saline containing a calcium chelator (Fig. 3a,d). In that case, pictures of final pigmentation state of experiments revealed fully open photophores (Fig. 3d).

These data together show that (*i*) IP_3_ does not trigger luminescence, while, conversely, it open photophore ILS in presence of calcium, (*ii*) melatonin does not require calcium to either trigger light emission or open the photophore ILS.

#### Calmodulin is involved in the light emission

**Figure 4:**
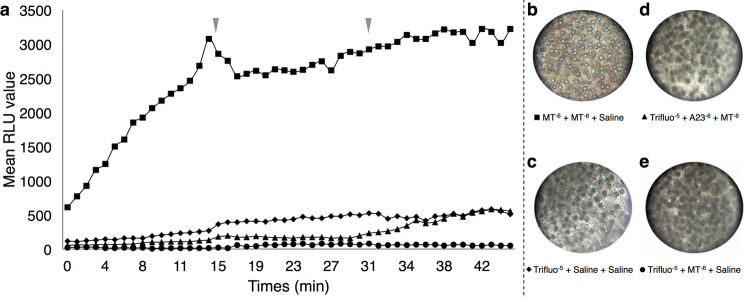
Ca^2+^-dependent calmodulin related lumino-pharmacological assays. (**a**) mean time-course evolution of light emission following the three application treatments showing that Trifluoperazine does not trigger light emission. Red arrowheads indicate the time of second and third drug applications. Final ventral skin patch pigmentation state after (**b**) MT 10^−6^ mol l^−1^ + MT 10^−6^ mol l^−1^ + Shark saline applications showing a classical melatonin-related open state of photophores; (**c**) Trifluoperazine 10^−5^ mol l^−1^ + Shark saline + Shark saline applications showing a close state of photophores; (**d**) Trifluoperazine 10^−5^ mol l^−1^ + A23187 10^−6^ mol l^−1^ + MT 10^−6^ mol l^−1^ applications showing a close state of photophores; and (**e**) Trifluoperazine 10^−5^ mol l^−1^ + MT 10^−6^ mol l^−1^ + Shark saline applications showing a close state of photophores, highlighting the need of intracellular Ca^2+^ input and calmodulin activity to open lanternshark photophores.

As already depicted by Claes and Mallefet (2009), melatonin application at 10^−6^ M triggers lanternshark luminescence and ILS opening that allow the light to come out of the photophore (Fig. 4a,b)^44^. Conversely, the application of a calmodulin inhibitor (trifluoperazine, 10^−5^ M^85^) followed by a MT treatment with (Fig. 4a,d) or without (Fig. 4a,e) an increase of calcium does not generate any light emission (Fig. 4a,d,e). The *in vivo* calcium increase was performed through the application of the calcium ionophore A23187. This result highlights the putative implication of the calmodulin in the regulation of the light emission. Control with an application of the calmodulin inhibitor followed by two applications of shark saline does not trigger any light emission (Fig. 4a,c). The pictures of the final pigmentation state of treated tissue samples revealed fully closed/dark photophores in the case of the calmodulin inhibitor alone or followed by a MT application (with or without calcium increase) (Fig. 4c–e).

So, calcium-dependent calmodulin is, here, demonstrate as essential to open the *E. spinax* ILS photophore.

#### Dynein is involved in the light emission

**Figure 5:**
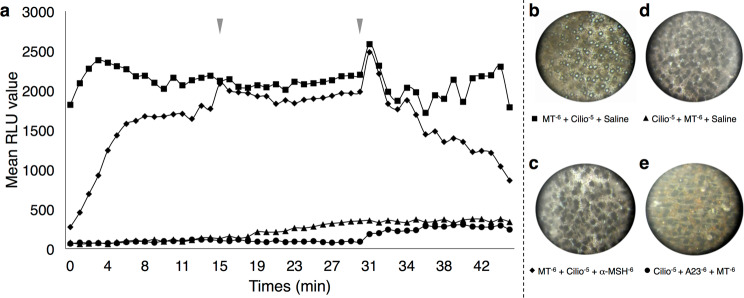
Minus-end intracellular motor dynein related lumino-pharmacological assays. (**a**) mean time-course evolution of light emission following the three application treatments showing that Ciliobrevin D before MT applications prevent light emission, while Ciliobrevin D after MT applications allow light to be emitted. Red arrowheads indicate the time of second and third drug applications. Final ventral skin patch pigmentation state after: (**b**) MT 10^−6^ mol l^−1^ + Ciliobrevin D 10^−5^ mol l^−1^ + Shark saline applications showing a classical melatonin-related open state of photophores; (**c**) MT 10^−6^ mol l^−1^ + Ciliobrevin D 10^−5^ mol l^−1^ + α-MSH 10^−6^ mol l^−1^ applications showing a classical α-MSH- related close state of photophores; (**d**) Ciliobrevin D 10^−5^ mol l^−1^ + MT 10^−6^ mol l^−1^ + Shark saline applications showing a close state of photophores; and (**e**) Ciliobrevin D 10^−5^ mol l^−1^ + A23187 10^−6^ mol l^−1^ + MT 10^−6^ mol l^−1^ applications showing a close state of photophores, highlighting the need of minus-end intracellular motor dynein to open lanternshark photophores.

Ciliobrevin D, an inhibitor of the dynein minus-end intracellular motor, was used at a concentration of 10^−5^ M to investigate the implication of dynein in the light emission process. Skin patches subjected to a first application of MT followed by a second application of ciliobrevin D emitted light (Fig. 5a–c). A rapid decrease of the light emission was then observed with a third application of α-MSH consistently with the observations of Claes and Mallefet (2009) (Fig. 5a)^44^. Conversely, an initial application of the ciliobrevin D, prevented the light emission with (Fig. 5a,e) or without (Fig. 5a,d) an increase of the *in vivo* calcium. The pictures of the skin patch pigmentation at the end of the experiment show (*i*) open photophores for the MT treatment followed by the ciliobrevin D treatment (Fig. 5b); (*ii*) closed photophores for the MT treatment followed by the ciliobrevin D and by an α-MSH application (Fig. 5c); (*iii*) closed photophores for both experiments starting with the ciliobrevin D treatment followed by MT application (Fig. 5d,e), even with an intracellular calcium increase (Fig. 5e). Control treatments with only ciliobrevin D or A23187 applications followed by shark saline applications did not trigger the light emission (data not shown). Therefore, these results highlight (*i*) the important role of dynein in the pathway regulating the opening of the photophore ILS, (*ii*) the non-implication of this later protein in the melanocortin regulation pathway.

#### Kinesin is involved in the light emission

**Figure 6:**
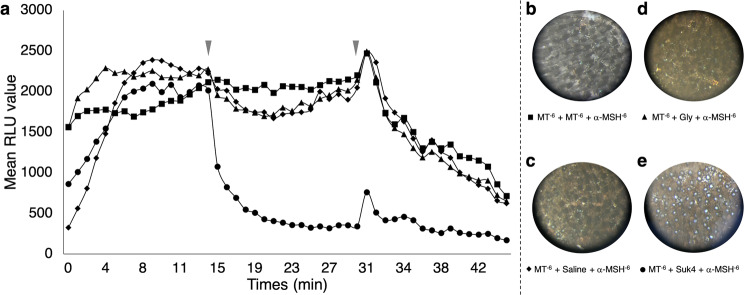
Plus-end intracellular motor kinesin-related lumino-pharmacological assays. (**a**) Mean time-course evolution of light emission following the three application treatments showing that SUK4 antibody inhibit light emission directly after application. Red arrowheads indicate the time of second and third drug applications. Final ventral skin patch pigmentation state after: (**b**) MT 10^−6^ mol l^−1^ + MT 10^−6^ mol l^−1^ + α-MSH 10^−6^ mol l^−1^ applications showing a classical α-MSH-related close state of photophores; (**c**) MT 10^−6^ mol l^−1^ + Shark saline + α-MSH 10^−6^ mol l^−1^ applications showing a classical α-MSH-related close state of photophores; (**d**) MT 10^−6^ mol l^−1^ + Glycerol 50% in shark saline + α-MSH 10^−6^ mol l^−1^ applications showing a close state of photophores; and (**e)** MT 10^−6^ mol l^−1^ + SUK4 antibody + α-MSH 10^−6^ mol l^−1^ applications showing an open state of photophores, highlighting the need of plus-end intracellular motor kinesin to close lanternshark photophores.

Conversely to the dynein, the kinesin is a plus-end intracellular motor which carries pigment vesicles from the nucleus periphery to the cell extremity. SUK4 antibody was used to inhibit the action of kinesin^86,87^. Starting with MT application, light emission was observed during the first 15 min (Fig. 6a). Luminescence was maintained after a second application with MT (Fig. 6a,b) or shark saline (Fig. 6a,c). Conversely, a rapid decrease of the light emission was shown with the secondary application of the SUK4 antibody (Fig. 6a). This rapid decrease of luminescence was not observed in the control treatment (*i.e*. secondary application of glycerol) (Fig. 6a,d).

Finally, a decrease of the emitted light was recorded for all experiments after a third drug, α-MSH, application (Fig. 6a). With the α-MSH last application, inhibiting the light emission, closure of photophores was expected (Fig. 6b–d). However, photogenic skin patch pre-treated with the kinesin inhibitor, SUK4, presented fully open photophores (Fig. 6e) similarly to the ones observed during the melatonin application alone (Fig. 4b). Control treatments with only SUK 4 or glycerol application followed by applications of shark saline did not trigger any light emission (data not shown).

Thus, kinesin is, here, demonstrate as main actor in the translocation of pigmented granules leading to the closure of the ILS photophore. Moreover, this protein is showed to also be involved in the light emission regulation.

## Discussion

By deciphering the molecular and cellular processes underlying the lanternshark counterillumination behaviour using molecular and pharmacological assays, this work highlights the interlinked pathways of bioluminescence, photoreception and pigmentation control within photophores. Our work, demonstrated the implication of (*i*) Es-Opn3 and (*ii*) actors involved in both MT and α-MSH/ACTH pathways on the velvet belly lanternshark bioluminescence and ILS cell pigment motions. Our results revealed the implication of Es-Opn3, MT, IP_3_, intracellular calcium, calcium-dependent calmodulin and dynein in the ILS cell pigment aggregation. Conversely, our results also highlighted the implication of an α-MSH/ACTH pathway involving kinesin to regulate the ILS cell pigment dispersion.

Based on previous pilot studies revealing an abundant and singular presence of Es-Opn3 within the photogenic ventral skin tissue^77,78^, the opsin absorbance spectrum was measured and revealed a blue-green absorption wavelength: between 410 and 490 nm, with a maximum value of 445 nm. This result is consistent with recent studies that have determined and characterized photoreceptive properties for several vertebrate Opn3 homologs (Table 1) such as fishes^88^ or other vertebrates^88,89^. The mosquito Opn3 (Mos-Opn3) was shown to form a bistable photopigment, absorb blue-green light, and activate Gi/o protein in a light-dependent manner^90–92^. Despite an increasingly number of newly-discovered opsin 3 homolog sequences both in invertebrates and vertebrates, the molecular properties of these proteins remain elusive with only a few studies describing the Gi/Go activation and the light-dependent cAMP modulation^90,92^. The measured Es-Opn3 absorption spectrum strongly suggests that lanternsharks can detect both environmental and their own light at the photophore level since a clear overlap with the intrinsic luminescence emission spectrum is observed.

**Table 1:** Known Opn3 maximum absorption wavelength from different metazoan species.

Species	Maximum absorption wavelength (nm)	References
Zebrafish	~465	^88^
Chicken	~470	^88^
Pufferfish	~470	^88^
Mosquito	~465	^90^

Our results show that deep blue and blue-green light exposures cause a clear modification of the IP_3_ intracellular level, adding evidence of a blue light extraocular photoreception at the level of the ventral skin (Fig. 2). No significant IP_3_ concentration change was observed for the red light or dark condition. No cAMP concentration change was observed during light exposure experiments neither in blue nor in red or dark conditions. These results are contradictory to what is known for the Mos-Opn3 literature as an efficient decrease of cAMP was observed for cells expressing Mos-Opn3 homolog^90,92^. Even if previous studies depicted an inefficient activation of Gq protein in Mos-Opn3 expressing cells^90^, our results suggested that Es-Opn3 would rather be linked to a Gq than a Gi/o protein, or that β/γ subunit of the Gi/o protein might be able to trigger an IP_3_ intracellular modulation and do not modify cAMP intracellular level. Therefore, Es-Opn3 is assumed to directly act through its specific G protein on the inositol lipid signalling system and trigger the activation of G protein-regulated phospholipase C. Further studies need to be conducted to precisely describe the downstream pathways of this deep-sea lanternshark specific opsin.

In addition to Opn3, the hormones controlling bioluminescence (MT, α-MSH, ACTH) are also shown to regulate skin pigmentation^6–16^. For this reason, we investigated the possible links between these proteins. Going further in the transduction pathways, pharmacological results demonstrated the interlinking of both bioluminescence, photoreception, and pigmentation in the light emission control. Our results (*i*) showed that melatonin does not require calcium to trigger light emission and (*ii*) highlight the involvement of the Ca^2+^-binding protein calmodulin and minus-end cellular motor dynein in the MT action way (Figs. 3–5). The classical MT pathway is related to the inhibition of the adenylate cyclase activity through the activation of a Gi α subunit protein^93–96^. The β/γ subunits of the Gi protein, release from the α subunit after the melatonin receptor activation, also display various effector actions such as the opening of calcium channels or the activation of specific phospholipases^97,98^.

**Figure 7:**
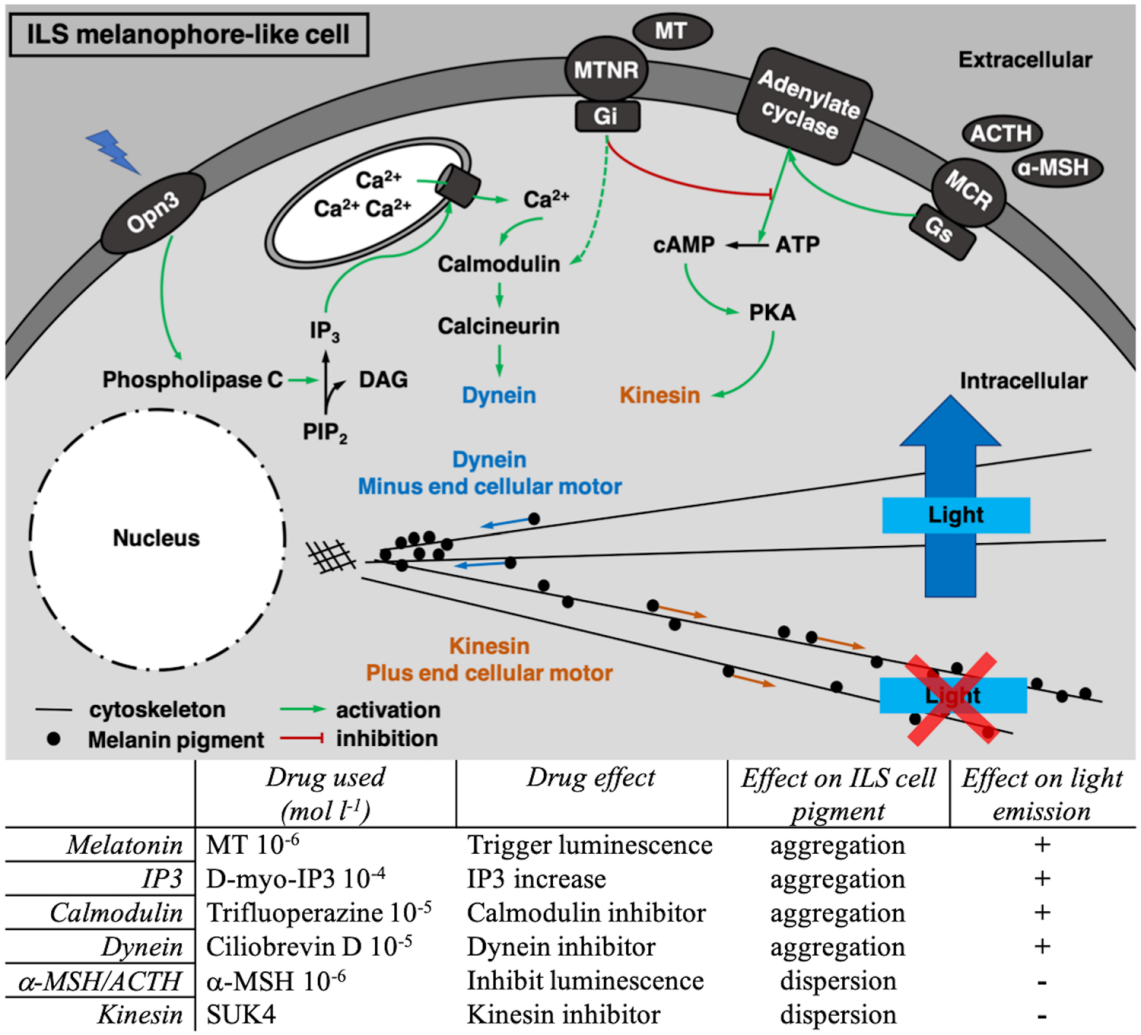
Schematic representation highlighting the different pathways involved in the physiological color change (pigment motion) in the ILS melanophore-like cells and implicated in the bioluminescence control of *E. spinax* (up); Pathways ending by kinesin plus-end cellular motor lead melanin granule dispersion (orange color) and prevent light to pass toward ILS (red cross), while pathways ending by dynein minus-end cellular motor lead to pigment aggregation and allow light to reach the photophore outside (blue arrow). A summary table of the different drug effects on both pigment motion and light emission is presented (down). ACTH, adrenocorticotropic hormone; ATP, adenosine triphosphate; cAMP, cyclic adenosine monophosphate; DAG, diacylglycerol; IP_3_, inositol triphosphate; MCR, melanocortin receptor; MT, melatonin; MTRN, melatonin receptor; MSH, melanocyte-stimulating hormone; PIP_2_, phosphoinositol bisphosphate; PKA, cAMP-dependent protein kinase A.

Pharmacological assays also highlighted that IP_3_ acts at the level of the ILS melanophore-like cells and is needed to aggregate ILS cell pigments. IP_3_ is already known as leading the aggregation of pigmented vesicles in fish and amphibian chromatophores^84,99^. However, IP_3_ increase does not directly trigger the luminescence of *E. spinax* (Fig. 3). In the same manner, our results demonstrate the necessity of calcium and the calmodulin activity to trigger the aperture of the ILS cells (Figs. 3, 4). Calcium is involved in the organelle motility in a huge variety of cell types, including fish chromatophores in which an increase of intracellular Ca^2+^ level triggers the aggregation of pigmentary organelles in the perikaryon^100–102^. Furthermore, studies demonstrated that the regulation of pigment motion involving Ca^2+^ is mainly provided by intracellular storage^103–105^. Other studies also highlighted the action of calmodulin activity on the cell pigmentation in various species (*i.e*. melanophores and erythrophores) where an increase of the Ca^2+^-binding protein calmodulin activity leads to the aggregation of pigmented vesicles in the nucleus periphery^103,106,107^. Although not studied in this work, the role of calcineurin has also been demonstrated in fish pigment vesicle aggregation and the protein is assumed to act in the IP_3_ transduction pathway regulating the aperture of the photophore ILS cells. Calcineurin mediates pigment aggregation in fish melanophores as a Ca^2+^/Calmodulin-stimulated phosphatase which dephosphorylates a 57 kDa protein, freeing cytoplasmic dynein^85^. Through the calcineurin phosphatase activity, previous studies depicted the involvement of melanophore-located cytoplasmic dynein as a minus-end cellular motor that carries pigmented granules/vesicles toward the nucleus periphery leading to more lighter melanophore cells and skin^12,108–110^. Following the pathway, our results demonstrated the necessary involvement of dynein to aggregate pigment and open the lanternshark photophores (Fig. 5). Combining all these pharmacological data with the literature supports a first model into which the ILS aperture occurs through the activation of dynein and the movement of pigmented granules allowing light to go out of the photophore. A potential pathway is, hence, suggested for the ILS aperture: (*i*) through the Es-Opn3 luminescence perception and activation, intracellular level of IP_3_ increases leading to (*ii*) the release of stored intracellular Ca^2+^ which activates (*iii*) the Ca^2+^-dependent calmodulin activity, which in turn, (*iv*) triggers the activity of calcineurin phosphatase stimulating (*v*) the cytoplasmic dynein. This later could, then, transport melanin granules from the cellular periphery toward the nucleus periphery and allow the outward light emission (Fig. 7).

Our data also decipher a second pathway involved in the photophore ILS closure and the inhibition of *E. spinax* light emission. Firstly, results clearly support the independency between the α-MSH pathway and dynein activity since ciliobrevin D dynein inhibition does not block the photophore closure by α-MSH application (Fig. 5c). By opposition with the first described cascade, this second pathway involves the cellular plus-end motor kinesin which is demonstrated to be needful to close the photophore but also to trigger the light emission (Fig. 6). Interestingly, when blocking kinesin activity with the SUK4 antibody^86^, (*i*) light emission was suppressed and (*ii*) ILS melanophore-like cell pigments remained fully aggregated even with later α-MSH treatment (Fig. 6). Here, the observed light emission suppression leads to assume the involvement of the kinesin to translocate granules containing potential bioluminescent reaction compounds or accessory activators of light reaction (*i.e*. cofactors) from the nucleus side of the photocyte to the other side (*i.e*. from vesicular to granular area^71,72^). Indeed, luminous reactions can involve either a luciferase/luciferin or a photoprotein in which a co-factor such as an ion is needed. Concerning the ILS pigment motion regulation, previous studies demonstrated the implication of various molecules in the α-MSH/ACTH and MCR pathway such as Gαs, adenylate cyclase and cAMP in the luminescence control of *E. spinax*^44,48,76^. Studies on metazoan melanophore granule dispersion highlighted the effector role of the cAMP-dependent protein kinase (PKA) in the MSH/MCR pathway in which the second messenger cAMP increases due to the adenylate cyclase up-regulation leading to the release of the catalytic subunit of PKA^7,88,111–113^). This kinase was shown to directly phosphorylate a granule-bound 53–57 kDa protein which leads to the activation of the specific heterotrimeric kinesin II as microtubule motor responsible for the pigment dispersion^7,114,115^. In addition to these previous researches, our data strongly suggest that this second pathway occurs in the ILS cells to close the photophore and prevent light to go out (Fig. 7). Therefore, data demonstrate that ILS cells pigment motion is under a dual and antagonist control by IP_3_/Ca^2+^ and cAMP pathways controlling the bidirectional granule movement in the melanophore-like cells, co-opted from skin pigmentation regulation in metazoan^103,116^.

Lanternsharks have a range of ways to control their luminescence and remained cryptic to avoid being spotted by underneath swimming predators. The mechanism allowing shallow water organisms to hide from predators through physiological colour change^1–4,6–16^, is here demonstrated to regulate the light emission of counterilluminating lanternsharks. This new luminescence control mechanism might also occur in other bioluminescent marine organisms displaying light organ associated melanophores for the counterillumination regulation such as cephalopods^117,118^ and bony fishes^119,120^.

## Conclusion

Using molecular and pharmacological assays, our results help to decipher some of the molecular pathways underlying the lanternshark counterillumination behaviour. This work highlights the functional interconnexion of bioluminescence, photoreception and pigment motion control within the photophores. Our data allow us to propose two interlinked pathways that regulate the pigment movement in specific melanophore-like cells within the ILS of the photophore and regulate the amount of light emitted by the lanternshark *E. spinax*. With clear overlapping between the lanternshark light emission wavelength and the Es-Opn3 absorption spectra, associated with the illumination assay and the pharmacological data, one of the transduction cascades starting with the absorption of blue-green light (probably the emitted bioluminescence) and resulting in the ILS pigment aggregation and, at the photophore level, the light passage is proposed. On the other side, α-MSH/ACTH known to inhibit light emission is described as acting on the transduction cascade leading to the pigment dispersion and, at the photophore level, avoidance of light emission. Finally, data also demonstrated that MT is involved in the ILS-cell pigmentation regulation by firstly downregulating α-MSH/ACTH transduction cascade, and secondly is implicated in the aperture of the photophore allowing light to go out of the photophore.

Evidence of the link between photoreception and photoemission in lanternshark is presented and highlights the implication of Es-Opn3 photopigment as a regulator of light emission through ILS cell pigment aggregation regulation. Although many other transduction cascades leading shark bioluminescence remain to be described, the present study highlight pathway parts regulating mechanically, through pigment bidirectional motion regulation, the light emission supervising the bioluminescence use for camouflage by counterillumination in the velvet belly lanternshark, *E. spinax*.

## Material and methods

### Fish & tissue collections

Twenty-four adult specimens of *E. spinax* were caught alive during a field session in May 2018 by longlines lowered at 220 m depth in the Raunefjord, Norway (60°169′ N; 05°089′ E)^44,48,71,72,121^. Specimens were directly placed in a 1 m^3^ tank filled with running fresh seawater (6 °C) and kept in a dark cold room at Espegrend Marine Station (Bergen University, Norway)^44,48,71,72,121^. Animal procedures were conducted in compliance with the Belgian national guidelines and in agreement with the European directive 2010/63/UE, under the approval of the Animal Ethics Committee of the Université catholique de Louvain in Louvain-la-Neuve. Sharks were treated according to the European regulation for animal research handling and euthanized following the local rules for experimental vertebrate care^73,76,77,121^. Sharks were sexed, measured and weighed before experimentation took place. Following Duchatelet *et al*. (2020), round shape piece of skin (6 mm diameter; ±100 mg of fresh tissue) were dissected from the ventral skin of sharks^44,48^. These skin patches, full of photophores, were rinsed in shark saline (292 mmol l^−1^ NaCl, 3.2 mmol l^−1^ KCl, 5 mmol l^−1^ CaCl_2_, 0.6 mmol l^−1^ MgSO_4_, 1.6 mmol l^−1^ Na_2_SO_4_, 300 mmol l^−1^ urea, 150 mmol l^−1^ trimethylamine N-oxide (TMAO), 10 mmol l^−1^ glucose, 6 mmol l^−1^ NaHCO_3_; total osmolarity: 1.080 mosmol; pH 7.7^122^) during half a day at 4 °c before pharmacological tests and light exposure tests happen. Sixteen sharks were used for the pharmacological experiment and eight sharks were used for the light exposure assay.

### Opn3 absorbance measurement

Recent studies highlight the presence of an opsin 3 colocalized with *E. spinax* photophores^77,78^. Following this research, the opsin 3 mRNA sequence was *in silico* extracted from the transcriptome, and cDNA was created. To increase the purification efficiency of the pigment, an Es-Opn3 deletion mutant was constructed having a shorter C terminus^83,90,123^. The cDNA of the C-terminal-truncated lanternshark Opn3 was generated from full-length cDNA. The C-termini of full length and C-terminal-truncated Es-Opn3 were tagged with the rho 1D4 epitope sequence (ETSQVAPA)^124^. The tagged Opn3 cDNAs were inserted between the Hind III and Eco RI sites of the pcDNA3.1 expression vector (GenScript USA inc.). The expression and purification of the Es-Opn3 were performed as previously described^123,125,126^. Opsin expression vectors were transfected into COS1 cells (a human embryonic kidney cell line) using the polyethyenimine (PEI) transfection method^123,125^. Transfected cells were collected two days post-transfection^123,125^. Opsin-based pigments were extracted with 1% dodecyl-ß-D-maltoside (DM) in HEPES buffer (pH 6.5) containing 140 mM NaCl and 3 mM MgCl_2_ (buffer A), after addition of 11-*cis* retinal. Extracted pigments were bound to 1D4-agarose gels, washed with 0.02% DM in buffer A and eluted with buffer A containing 0.02% DM and c-terminal peptide of bovine rhodopsin as described^123,125^. The absorption spectra of the opsin-based pigments were recorded at 4 °C by using a Shimadzu UV2450 spectrophotometer^123,125^.

### The implication of Es-Opn3 in light perception

To see the implication of light perception at the level of the light organ, skin patches were placed under different wavelengths during different times. In brief, after the 30 min rinses in shark saline, two skin patches of each shark were placed in each well of a 12 wells plate. These plates were placed at 20 cm of the light sources. The light sources used to irradiate the skin patches are a blue/violet LED (415 nm), blue light LED (480 nm) and red light LED (630 nm) (Hydong, Shenzhen, China). During stimulation, skin patches were bathed in 3 mL of shark saline buffer. The blue light LED (480 nm) was selected to mimic the shark light emission (~486 nm), deep blue light (415 nm) was selected to see if shark skin patches can perceive shorter wavelength, and, finally, red light (630 nm) was selected to see the specificity of the shark Opn3. Two skin patches of each shark were maintained in shark saline in a fully dark condition to display dark state control. Skin patches were exposed to light during 15, 30 or 45 min. Thus, two patches of one shark are subjected to one wavelength during one period. After each period, skin patches were removed and directly frozen at −80 °C. One skin patch was devoted to inositol triphosphate (IP_3_) concentration level assay and the second, to the cyclic adenosine monophosphate (cAMP) concentration level assay. Each skin patches were weighted before to be homogenized in phosphate buffer saline with 0.02% Triton X-100 (Sigma) thanks to a grinder (T10 Basic UltraTurrax, IKA) on ice, and then centrifuged at 15000 rpm for 5 min at 4 °C. Supernatants were recovered, aliquoted and stored at −20 °C until experiments took place.

### IP_3_ assay

First skin patches of each shark treated according to light and time exposure were subjected to an IP_3_ concentration assay [IP_3_ (Inositol Triphosphate) ELISA Kit, E-EL-0059, Elabscience Biotechnology, USA] to determine the potential modification of the IP_3_ concentration. Briefly, supernatants of each treatment were placed in a well of a pre-coated with IP_3_ 96-wells plate and treated following the IP_3_ competitive-ELISA manufacturer’s instructions. Colourimetric results [optical density (O.D.)] were measured by a spectrophotometer (SpectraMax 190, Molecular Devices, San Jose, CA, USA) coupled with SoftMax Pro 6.5.1 software (Molecular Devices; https://www.moleculardevices.com/). The concentration of IP_3_ in the samples was determined by comparing the O.D. of the samples to the IP_3_ standard curve. Results are expressed in picogramme of IP_3_ per millilitre per gramme of tissue (pg ml^−1^ per g of tissue).

### cAMP assay

cAMP concentrations were measured on the second skin patches of each shark treated according to light and time of exposure thanks to cAMP-Glo Assay kit (V1501, Promega, Madison, WI, USA) following Duchatelet *et al*.^48^. Briefly, supernatants of each treatment were placed in a well of a 96-wells plate and treated according to the kit manufacturer’s instructions. Luminescence induced during the assay was recorded thanks to a microplate luminometer (Berthold MPL12/Orion; Pforzheim, Germany) coupled with the Berthold simplicity software (http://www.titertekberthold.com/). According to cAMP-Glo Assay kit linear regression manufacturer standard, results are expressed in cAMP concentration and refine standardized in cAMP concentration per square centimetre (µM cm^−2^).

### Pharmacological strategies

To decipher the different steps of the pathway involved in the extraocular light perception and the hormonal control of the light emission, luminometric assay after drug application blocking or triggering step by step transduction events were done. Investigations were done to decipher the implication of (*i*) IP_3_ and calcium; (*ii*) Ca^2+^-dependent calmodulin; (*iii*) cytoplasmic dynein; and (*iv*) kinesin in the transduction pathways leading to light emission control. The following drugs were employed: melatonin (M5250, Sigma) activates the light production in lanternshark^44^; α-MSH (M41135, Sigma) inhibit the light production by lanternshark photophores^44^; D-myo-Inositol 1,4,5-tris-phosphate (I9766, Sigma) serves to manually increase the IP_3_ level in the skin patches; BAPTA (1,2-Bis(2-aminophenoxy)ethane-N,N,N’,N’-tetraacetic acid, 14513, Sigma) is a calcium chelator; calcium ionophore A23187 (C7522, Sigma) trigger an increase of calcium in the light organ cells; trifluoperazine hydrochloride (T2000000, Sigma) inhibit the Ca^2+^-dependent calmodulin activity; cytoplasmic dynein inhibitor, ciliobrevin D (250401, Sigma) acts as a reversible and specific blocker of AAA + ATPase motor cytoplasmic dynein; and antibody against kinesin heavy chain (SUK4, Developmental studies, Hybridoma Bank, University of Iowa) inhibit the kinesin driven microtubule motility^86^. Glycerol was used as control for SUK4 experiments as the antibody is diluted in this substrate. Used concentrations were extracted from literature and are summarized in Table 2.

**Table 2:** Used concentrations during pharmacological tests extracted from previous literature.

Drugs	Concentration (mol l^−1^)	References
Melatonin	10^−6^	^44,48^
α-MSH	10^−6^	^44,48^
D-myo-IP_3_	10^−4^	^84^
BAPTA	10^−5^	^84^
A23187	10^−6^	^100,116^
Trifluoperazine	10^−5^	^85^
Ciliobrevin D	10^−5^	^127^

Patches of ventral skin were placed in 96-well plates and subject to various treatments (Table 3). A total of three applications of drugs occurred during one experiment. The first application was done at the beginning of the experiment, the second application at 15 min and the last one at 30 min. Data were recorded for 45 min thanks to a microplate luminometer (Berthold MPL12/Orion; Pforzheim, Germany) coupled with the Berthold simplicity software.

**Table 3:** Summary of the application steps used during the pharmacological test.

Target in the pathway	First application	Second application	Third application
Inositol triphosphate	D-*myo*-IP_3_	Shark saline	Shark saline
	BAPTA	D-*myo*-IP_3_	Shark saline free Ca^2+^
	BAPTA	Melatonin	Shark saline free Ca^2+^
Calmodulin	Trifluoperazine	Shark saline	Shark saline
	Trifluoperazine	A23187	Melatonin
	Trifluoperazine	Melatonin	Shark saline
	Melatonin	Melatonin	Shark saline
Dynein	Ciliobrevin D	Shark saline	Shark saline
	Ciliobrevin D	A23187	Melatonin
	Melatonin	Ciliobrevin D	α-MSH
	Melatonin	Ciliobrevin D	Shark saline
	Ciliobrevin D	Melatonin	Shark saline
Kinesin	Melatonin	SUK4 antibody	α-MSH
	Melatonin	Glycerol 50%	α-MSH
	Melatonin	Shark saline	α-MSH
	Melatonin	Melatonin	α-MSH

### Pigmentation visualization

At the end of each lumino-pharmacologic assay, a picture of each treated ventral skin patch has been taken to evaluate the pigmentation state. Pictures were taken thanks to a Lumix DMC-FZ300 camera (Panasonic Corporation, Osaka, Japan).

### Statistical analyses

All analyses [ANOVA, post-hoc Tukey tests] were performed with the software JMP pro v.14 (SAS Institute Inc., Cary, NC, 1989–2007). The Gaussian distribution (Shapiro test) and the homoscedasticity (Levenee test) were obtained for all analyses after logarithm transformation allowing the use of parametric tests. ANOVA was used to show significant differences between groups while post-hoc Tukey tests allowed the different clusters to be distinguished.

## Supplementary information

Supplementary information.

## Data Availability

The datasets generated during and/or analysed during the current study are available from the corresponding author on reasonable request.
