# Supplementary material for: From extraocular photoreception to pigment movement regulation: a new control mechanism of the lanternshark luminescence
Source: Sci Rep. 2020 Jun 23;10:10195. doi: 10.1038/s41598-020-67287-w (PMC7311519; doi:10.1038/s41598-020-67287-w)

From extraocular photoreception to pigment movement regulation: a new control mechanism of the lanternshark luminescence

Scientific Reports

Laurent Duchatelet[1*], Tomohiro Sugihara[2], Jérôme Delroisse[3], Mitsumasa Koyanagi[2], René Rezsohazy[4], Akihisa Terakita[2], Jérôme Mallefet[1]

[1]Université Catholique de Louvain (UCLouvain), Marine Biology Laboratory, Earth and Life Institute, 3 Croix du Sud, Louvain-La-Neuve 1348 - Belgium

[2]Osaka City University, Department of Biology and Geosciences, Graduate School of Science, Osaka 558-8585 – Japan

[3]Université de Mons (UMONS), Biology of Marine Organisms and Biomimetics, Research Institute for Biosciences, 23 Place du Parc, 7000 Mons, Belgium

[4]Université Catholique de Louvain (UCLouvain), Animal Molecular and Cellular Biology, Louvain Institute of Biomolecular Science and Technology, 5 Croix du Sud, Louvain-la-Neuve 1348 - Belgium

Corresponding authors: L. Duchatelet: laurent.duchatelet@uclouvain.be, +32 (0)10 47 34 75

Supplementary Figure S1 Graphical view of the *E. spinax* opsin 3 absorption spectra obtained by expression and purification of them using mammalian cultured cells. Absorption spectra of the full-length Es-Opn3 (upper) and the C-terminal-truncated Es-Opn3 (lower)

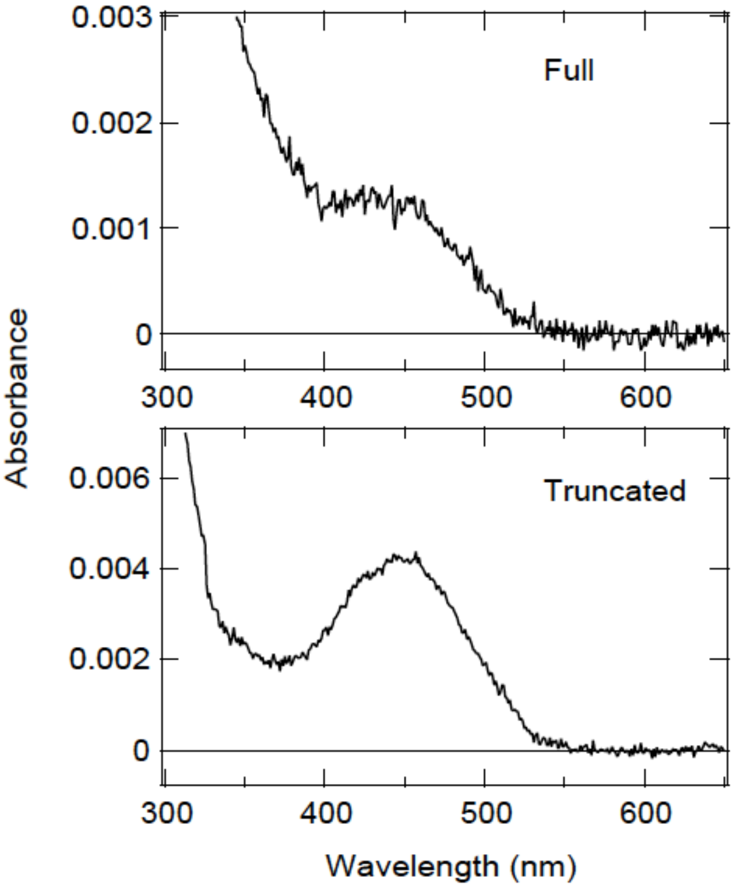

Supplement: Supplementary file 1 — Supplementary information. [file 41598_2020_67287_MOESM1_ESM.pdf]
